# Impact of lateral positioning on upper airway morphology in sedated children under five

**DOI:** 10.1007/s12519-025-00910-w

**Published:** 2025-04-29

**Authors:** Hui Li, Xuan Jia, Hui Ye, Jia-Wei Liang, Wen-Li Zhao, Ping Cui, Yaqi Sun, Deng-Ming Lai, Qiang Shu, Yue Jin, Guo-Hao Xie, Xiang-Ming Fang

**Affiliations:** 1 Department of Anesthesiology, The First Affiliated Hospital, School of Medicine, Zhejiang University, Wenyixi Road 1367, Hangzhou, 311121 China; 2 Department of Radiology, Children’s Hospital, Zhejiang University School of Medicine, National Clinical Research Center for Child Health, Hangzhou, 310003 China; 3 Children’s Hospital, Zhejiang University School of Medicine, National Clinical Research Center for Child Health, Hangzhou, 310003 China; 4 Department of Hematology, Children’s Hospital, Zhejiang University School of Medicine, National Clinical Research Center for Child Health, Hangzhou, 310003 China; 5 Department of Neonatal Surgery, Children’s Hospital, Zhejiang University School of Medicine, National Clinical Research Center for Child Health, Hangzhou, 310003 China; 6 Department of Thoracic and Cardiovascular Surgery, Children’s Hospital, Zhejiang University School of Medicine, National Clinical Research Center for Child Health, Hangzhou, 310003 China; 7 Department of Anesthesiology, Children’s Hospital, Zhejiang University School of Medicine, National Clinical Research Center for Child Health, Hangzhou, 310003 China

**Keywords:** Children Lateral position Three-dimensional model, Under 5 years old, Upper airway

## Abstract

**Background:**

The upper airway morphology in children varies with age and body position. This study aimed to analyze the impact of lateral positioning on the upper airway of sedated children under five.

**Methods:**

This retrospective study included pediatric patients who underwent MRI in both the supine and lateral positions at Children’s Hospital, Zhejiang University School of Medicine. Upper airway morphology was reconstructed using 3D Slicer software. Python was employed to estimate cross-sectional areas via pixel analysis. The narrowest cross-sectional area, minimal transverse and anteroposterior diameters, airway length, and airway volume were measured and stratified by age for subgroup analysis.

**Results:**

In sedated children under 5 years old and when compared to the supine position, lateral positioning increased minimal transverse diameter by 18.70% (*P* = 0.001), narrowest cross-sectional area by 49.21% *(P* < 0.001), anteroposterior diameter by 25.54% (*P* < 0.001), airway volume by 65.64% (*P* < 0.001), and airway length by 11.93% (*P* < 0.001). In all subgroups, lateral positioning significantly increased the narrowest cross-sectional area, airway length, and airway volume. However, minimal anteroposterior diameter in the 1-to 3-year age group and minimal transverse diameter in the 3 -to 5-year age group tended to increase in the lateral position but did not reach statistical significance.

**Conclusions:**

Lateral position significantly enlarges the upper airway in sedated children under five. These findings support using lateral position to enhance airway patency in younger patients.

**Graphical abstract:**

**Figure:**
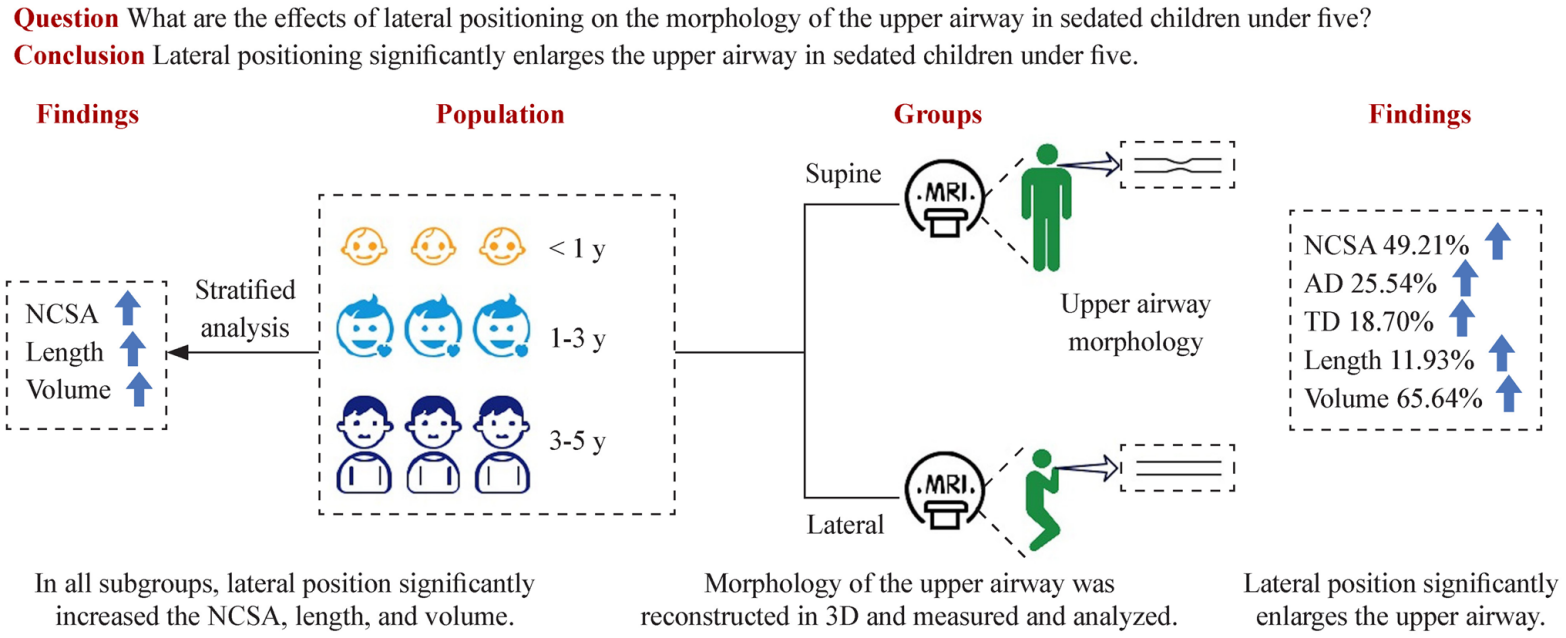
NCSA narrowest cross-sectional area, AD anteroposterior diameter, TD transverse diameter

## Introduction

Age-based anatomical and morphological changes in the larynx have vital clinical implications for airway management. In infants, the larynx is funnel-shaped, with the narrowest portion at the near-circular cricoid cartilage; this later transitions into a more cylindrical adult airway [1]. Significant developmental differences exist in airway anatomy and function between children and adults, particularly in children under five. In these children, their upper airway dimensions are primarily influenced by a large tongue, increased lymphoid tissue mass, and adenoid hypertrophy. Because of the narrowness of the pediatric larynx and glottis, even slight inflammation or edema can lead to breathing difficulties. Additionally, unique anatomical characteristics in pediatric patients—such as a shorter neck, a proportionally larger head-to-body ratio, and a prominent occiput—predispose them to excessive neck flexion. This alignment of the oral, laryngeal, and tracheal axes significantly increases the risk of upper airway obstruction in the supine position. By 6**–**8 years of age, the pediatric airway closely resembles that of an adult [2]. Understanding the upper airway morphology in children under five is helpful in the prevention, management, and treatment of airway events.

Magnetic resonance imaging (MRI) is an essential tool in pediatric imaging, offering detailed, high-resolution visualization of soft tissues without ionizing radiation. This is making it particularly suitable for children [3]. However, MRI is time-consuming and sensitive to motion artifacts. To minimize movement and obtain high-quality images, sedation or anesthesia is often required [4]. Sedation/anesthesia has been identified as an independent risk factor for safety [5], with 80% of complications related to adverse airway or respiratory events [6, 7]. A retrospective study on sedation/anesthesia for pediatric MRI reported that mild desaturation (SpO_2_ of 80%–89%) occurred in 4.22% of cases, while severe hypoxia (SpO_2_ of < 70%) occurred in 0.44% [8]. The unique morphological characteristics of the pediatric airway, combined with relaxation of upper airway muscles under sedation, contribute to airway collapse and hypoxia [9]. Parameters such as cross-sectional area and airway volume are commonly used to assess airway patency [10, 11]. Accurately describing the morphological of the upper airway in children under five using MRI has the potential to identify improved airway management strategies; a significant challenge for physicians.

Previous studies focused on adults have revealed that positional therapy effectively improves pharyngeal patency. In these studies, lateral positioning has been shown to resolve upper airway obstruction caused by the collapse of specific structures within the airway [12, 13]. The current study aimed to analyze morphological changes associated with the lateral position in the upper airways of children under five. We have studied children undergoing MRI and interrogated image-based three-dimensional (3D) reconstructions.

## Methods

**Fig. 1:**
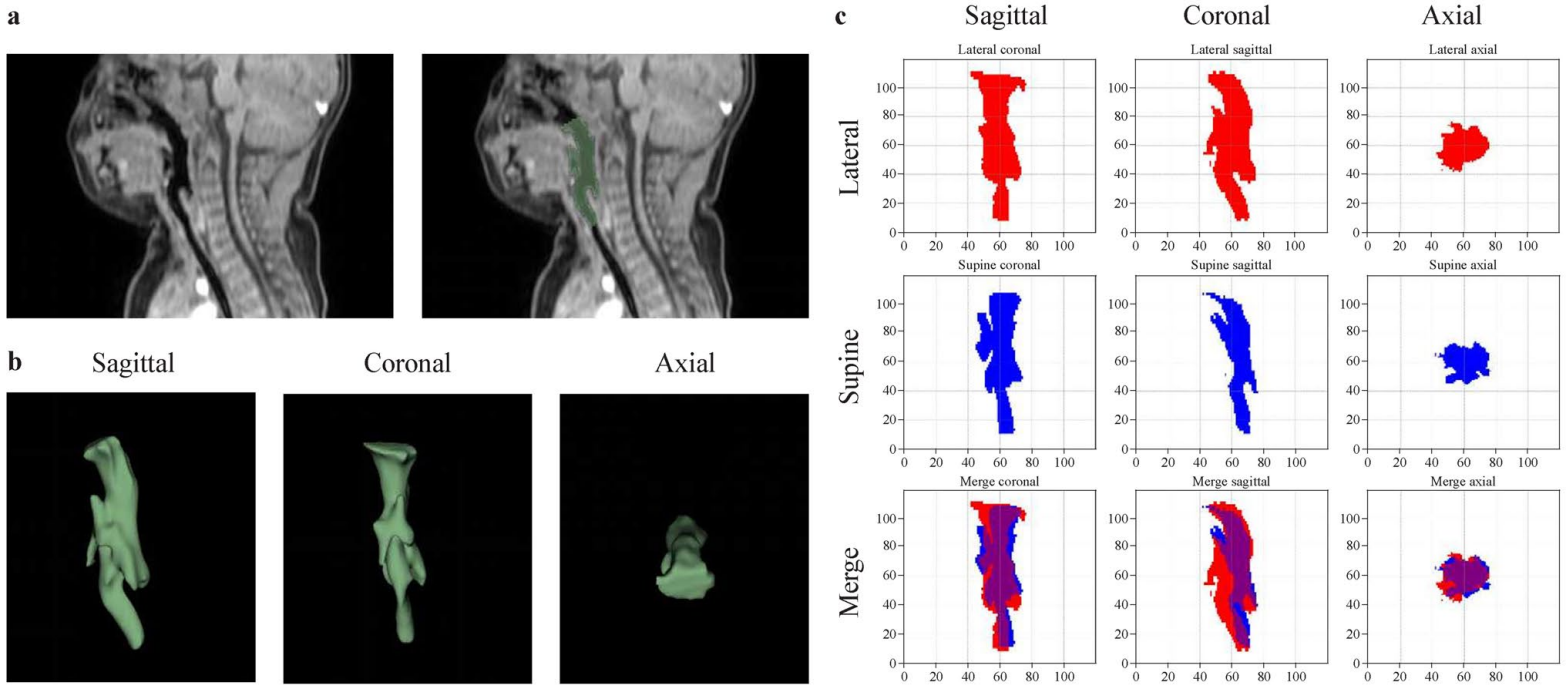
Upper airway 3D reconstruction and orthographic projections of the upper airway model. **Panel a**: The left image shows the sagittal MRI scan, while the right image shows the measured range of the upper airway; **Panel b**: A 3D-reconstructed stereoscopic image, displaying sagittal, coronal, and axial views; **Panel c**: Orthographic projections of the upper airway 3D model in the coronal, sagittal, and axial planes. The red and blue areas represent the lateral and supine projections of the 3D model, respectively, merged into one image for comparison

The study protocol was reviewed and approved by Ethics committee of Children’s Hospital, Zhejiang University School of Medicine (2023-IRB-0152-P-01) and was performed in accordance with the Declaration of Helsinki. The requirement for informed consent was waived due to the retrospective nature of the study. However, written consent for the publication of the MRI image in Fig. 1A has been obtained from the parent of the participant. The study was registered in the Chinese Clinical Trial Register (ChiCTR2400079840).

### Patients and MRI examinations

We searched the radiology database and conducted a retrospective review of pediatric patients under 5 years old who underwent MRI scanning for clinical purposes at Children’s Hospital, Zhejiang University School of Medicine between May 2020 and November 2023. The inclusion criteria were as follows: MRI scans that included both lateral and supine position scans, performance of MRI scan under sedation, and inclusion of the upper airway region in the MRI scan. Patients were excluded if their MRI scans were conducted in only the lateral or supine position.

Children were sedated before the scan using 0.5 mg/kg midazolam oral solution and 2 μg/kg dexmedetomidine nasal drops. If depth of sedation was insufficient, 6%–8% sevoflurane was administered for 3–5 minutes. The Modified Observer’s Assessment of Alertness and Sedation (MOAA/S) scale was used to evaluate the depth of sedation. MRI examinations began when the MOAA/S score was ≤ 2, indicating that the children did not respond to loud calls.

All MRI scans were performed using a Philips Prodiva CX 1.5T scanner. The routine MRI protocol included the T1-3D_mDixon sequence of the oropharyngeal region in sagittal views with a slice thickness of 2.5 mm and a 1.25-mm gap. Transverse section images (T1w, T1ip, T1op, and T1f) were analyzed, and coronal images were obtained using the maximum intensity projection post-processing method.

### Upper airway measurement and modelling

Upper airway geometries were segmented from the MRI scans using 3D-Slicer software (version 5.6.1) [8]. MRI data were imported into the software and a specific threshold was applied. The region-growing technique was then used to segment the upper airway structures, enabling extraction and reconstruction of airway morphology. Measurements of the upper airway were performed by a professional surveyor using the reconstructed 3D models.

Recorded data included patient demographics; age, sex, height, weight, and disease diagnosis. The narrowest cross-sectional area, the minimal transverse and anteroposterior diameters, and the volume and length of the upper airway in both the lateral and supine positions were measured, calculated, and analyzed.

### Upper airway 3D modelling

We used Python (version 3.12.7) to read segmentation images in seg.nrrd format using the nrrd package. Image orientation was adjusted using the SciPy package to align the airway axis with the Z-axis. We used the NumPy package to create two-dimensional (2D) projection plots and counted the pixels along the cross-sectional plane to estimate the projected area. Finally, the processed data were visualized using the Matplotlib package.

### Statistical analysis

Data analysis was conducted using SPSS statistical software version 25 (IBM Corp., Armonk, NY, USA). For normally distributed data, descriptive statistics are presented as mean ± standard deviation. Differences between paired groups were analyzed using the paired *t*-test. For data that do not follow a normal distribution, the median (interquartile range) was used for description, and differences between paired groups were assessed using the Wilcoxon signed-rank test. A *P*-value of < 0.05 was considered statistically significant.

## Results

### Patient information

There was a total of 24 patients in this study including 15 girls and 9 boys aged 0 to 5 years. The youngest patient was two months of age and the eldest was 4 years and 11 months. Weights ranged from 4.50 to 17.20 kg with an average of 11.35 ± 3.33 kg. Heights ranged from 55.00 to 106.00 cm with an average of 83.84 ± 13.11 cm. Clinical and demographic details are presented in Table 1.

**Table 1:** Patients’ characteristics

ID	Sex	Age	Height (cm)	Weight (kg)	Diagnosis
1	F	3Y3M	94.00	14.50	Neuroblastoma
2	F	11M	73.30	7.50	Nephroblastoma
3	M	4Y8M	106.00	16.40	Myofibroblast sarcoma
4	M	1Y9D	78.00	14.00	Myocarditis
5	F	1Y1M	73.00	8.30	Neuroblastoma
6	M	10M	73.00	8.30	Mediastinal neuroblastoma
7	F	3M	63.00	6.80	Teratoma
8	F	3Y1M	92.00	13.90	Vitelline sac tumor
9	F	10M	74.00	8.90	Neuroblastoma
10	F	4Y8M	101.00	15.20	B lymphoblastic leukemia
11	F	4Y2M	93.50	12.20	Neuroblastoma
12	M	2M	55.00	4.50	Infantile hemangioendothelioma
13	M	3Y9D	99.50	17.20	Fibroblast hyperplasia
14	F	1Y5M	80.00	10.30	Malignant germ cell tumor
15	M	11M	85.00	10.80	Neuroblastoma
16	M	4Y11M	101.00	15.00	Neuroblastoma
17	F	2Y3M	89.00	11.50	Ganglion cell neuroblastoma
18	F	3Y27D	97.50	13.80	Adrenocortical carcinoma
19	F	3Y6M	96.00	14.50	Neuroblastoma
20	F	2Y7Y	81.30	11.40	Ganglion cell neuroblastoma
21	M	1Y7M	82.00	10.00	Retroperitoneal neuroblastoma
22	F	7M	74.00	8.50	Duplicate kidney and hydronephrosis
23	M	9M	75.00	10.25	Neuroblastoma
24	F	1Y8M	76.00	8.60	Nephroblastoma

*Y* year, *M* month, *D* day, *M* male, *F* female

### 3D reconstruction and orthographic projections of the upper airway model

MRI revealed that the upper airway extended from the posterior edge of the hard palate to the level of the horizontal cricoid cartilage in the subglottic areais illustrated in Fig. 1a. The 3D reconstructed stereoscopic image is shown in Fig. 1b, is presented with sagittal, coronal, and axial views. Figure 1c displays orthographic projections of the 3D model of the upper airway filling in the coronal, sagittal, and horizontal planes. Red represents the projected area of the 3D model in the lateral position, while blue represents the projected area in the supine position. To clearly visualize differences between the supine and lateral projection images, both were merged onto a single image for comparative analysis.

**Fig. 2:**
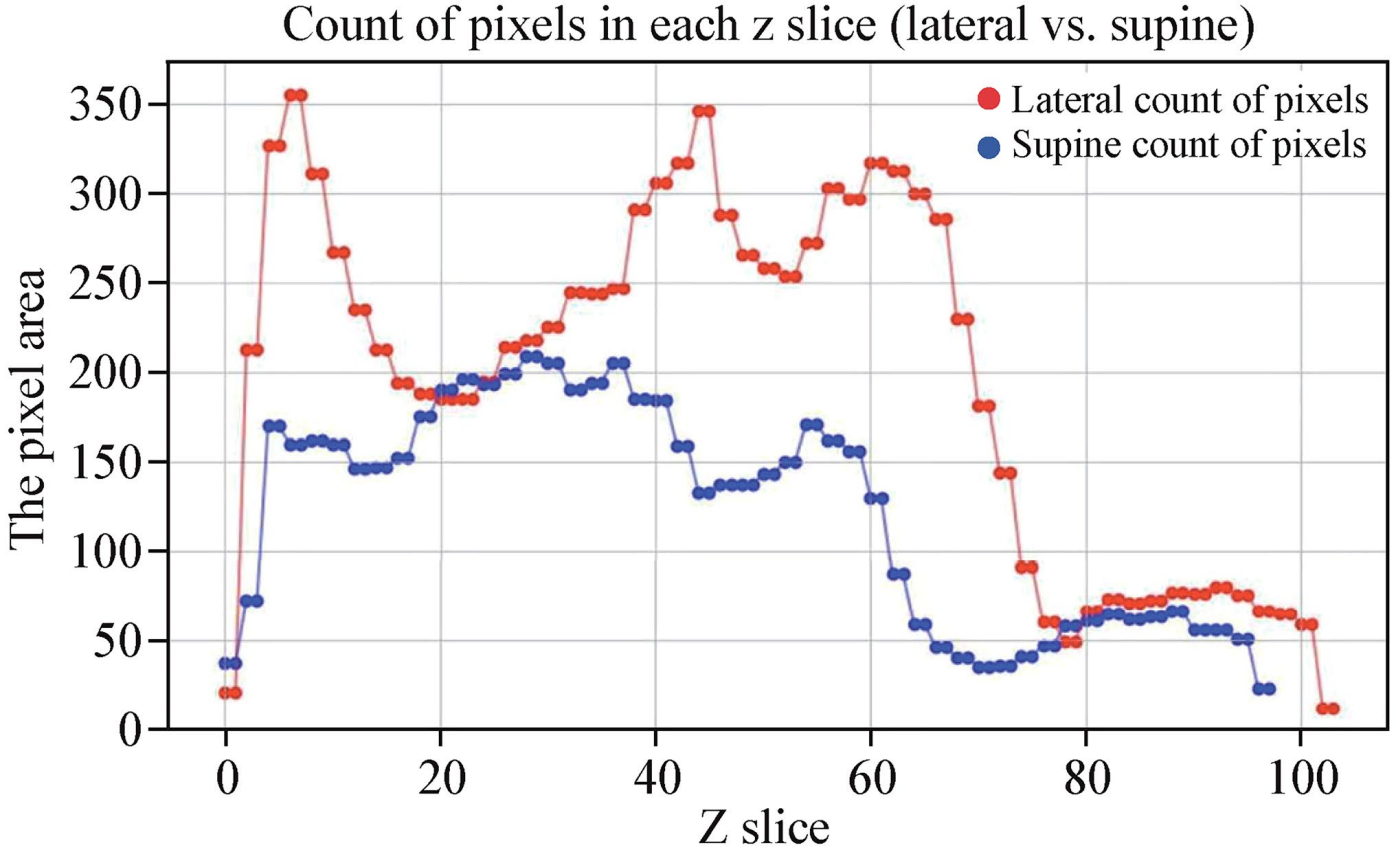
Pixel area of each cross-sectional slice of upper airway for supine and lateral positions. The horizontal axis represents the Z Slice, corresponding to each cross-sectional slice of the upper airway along its longitudinal axis. The vertical axis represents the pixel area of each respective Z Slice for one subject. The minimum Z Slice value corresponds to the upper edge of the upper airway, while the maximum value marks the lower edge of the upper airway

Cross-sectional slices of the upper airway were taken at equal intervals from top to bottom. The area corresponding to each slice was quantified in pixels. Pixel area of each cross-sectional slice was recorded to intuitively reflect changes in the cross-sectional area of the upper airway along the longitudinal axis in both the lateral and supine positions for a single subject (Fig. 2).

### Multidimensional measurements of the upper airway

**Fig. 3:**
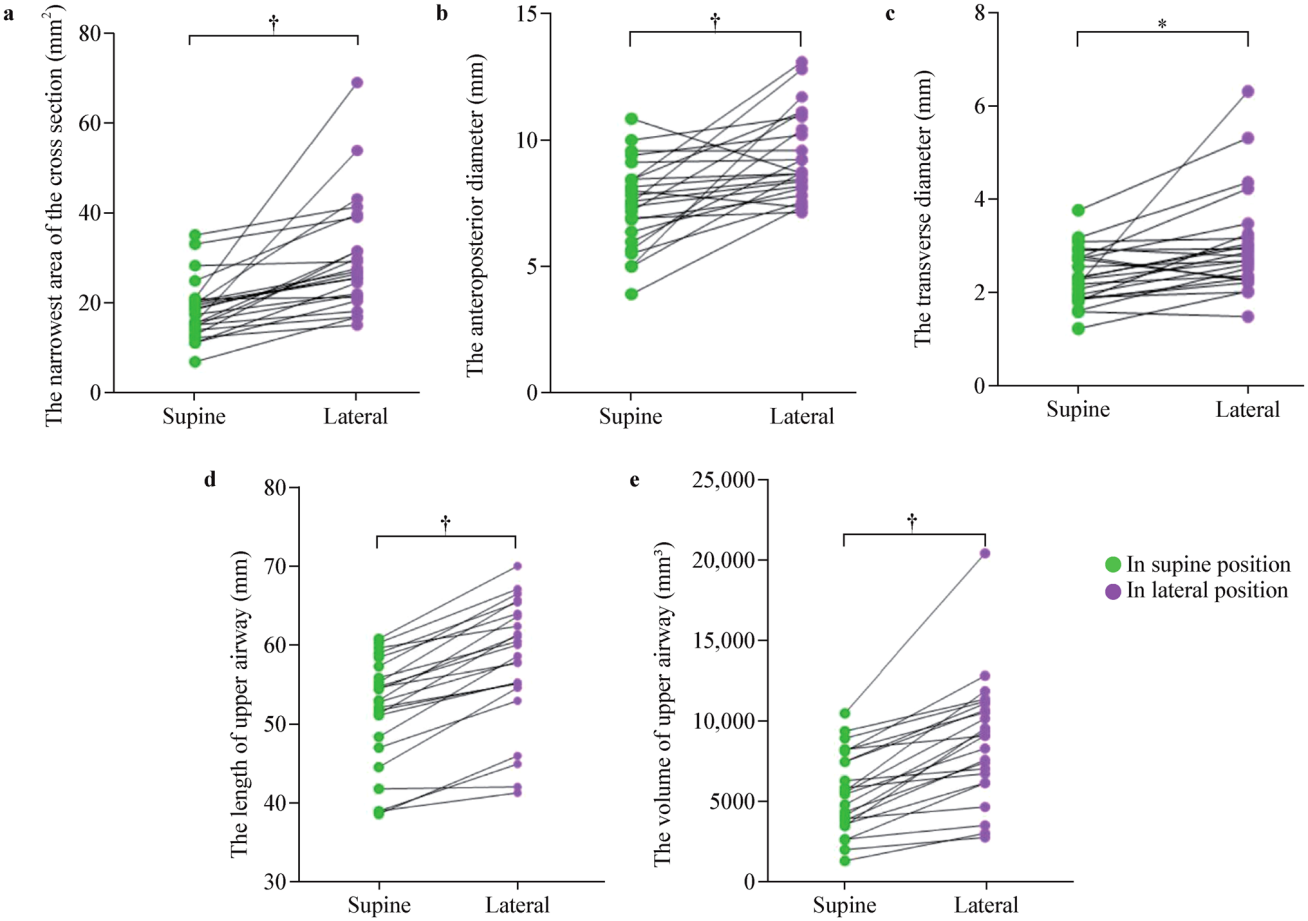
Comparative multidimensional measurements of upper airway between supine and lateral positions. Comparison of **a** narrowest cross-sectional area, **b** minimal anteroposterior diameter, **c** minimal transverse diameter, **d** length, and **e** volume between the supine and lateral positions. *P < 0.01, ^†^*P* < 0.001

To more intuitively illustrate morphological changes of the upper airway, we plotted point-to-point connection diagrams for measurements of each child in the supine and lateral positions (Fig. 3). In the lateral position, remarkable morphological alterations occurred in the upper airway. The narrowest cross-sectional area increased by 49.21% [17.82 (14.00–20.56) vs. 26.59 (21.20–37.08) mm^2^] (*P* < 0.001; Table 2 and Fig. 3a). The minimal anteroposterior diameter grew by 25.54% (7.44 ± 1.73 vs. 9.34 ± 1.72 mm) (*P* < 0.001; Table 2 and Fig. 3b). In addition, minimal transverse diameter expanded by 18.70% [2.30 (1.88–2.77) vs. 2.73 (2.29–3.23) mm] (*P* = 0.001; Table 2 and Fig. 3c). Moreover, length of the upper airway lengthened by 11.93% (51.81 ± 6.86 vs. 57.99 ± 7.91 mm) (*P* < 0.001; Table 2 and Fig. 3d). Notably, the volume of the upper airway augmented by 65.64% [5472.95 (3608.40–7929.20) vs. 9065.30 (6267.23–10976.19) mm^3^] (*P* < 0.001; Table 2 and Fig. 3e). Collectively, these changes have suggested potential physiological implications for related physiological functions and clinical applicability.

**Table 2:** Comparative multidimensional measurements of upper airway between lateral and supine positions

Measurement parameters	Supine position (*n* = 24)	Lateral position (*n* = 24)	*P* value
The narrowest cross-sectional area (mm^2^)	17.82 (14.00–20.56)	26.59 (21.20–37.08)	< 0.001
The transverse diameter (mm)	2.30 (1.88–2.77)	2.73 (2.29–3.23)	0.001
The anteroposterior diameter (mm)	7.44 ± 1.73	9.34 ± 1.72	< 0.001
The length of upper airway (mm)	51.81 ± 6.86	57.99 ± 7.91	< 0.001
The volume of the upper airway (mm^3^)	5472.95 (3608.40–7929.20)	9065.30 (6267.23–10976.19)	< 0.001

*P* < 0.05 indicates significance

### Stratified analysis by age

To explore the effect of body position on upper airway morphology at different stages of airway development, we categorized the patients into three age groups (0 - 1 year, 1 - 3 years, and 3 - 5 years) and performed a subgroup analysis (Table 3). Narrowest cross-sectional area, airway length, and airway volume were significantly greater in the lateral position than in the supine position across all subgroups. However, minimal anteroposterior diameter in the 1-to 3-year age group (7.36 ± 2.16 vs. 8.10 ± 0.82 mm, *P* = 0.376) and minimal transverse diameter in the 3- to 5-year age group [2.74 (2.31–3.09) vs. 3.16 (2.36–4.24) mm, *P* = 0.173)] tended to increase in the lateral position but did not reach statistical significance.

**Table 3:** Stratified analysis by age

Age groups	Measurement parameters	Supine position (*n* = 24)	Lateral position (*n* = 24)	*P* value
Less than 1 y old	The narrowest cross-sectional area (mm^2^)	12.70 (10.97–15.61)	24.37 (20.33–31.30)	0.012
	The transverse diameter (mm)	1.87 (1.62–2.29)	2.52 (2.21–2.67)	0.012
	The anteroposterior diameter (mm)	6.65 ± 1.22	9.44 ± 1.92	0.002
	The length of upper airway (mm)	46.49 ± 6.54	52.48 ± 9.09	0.002
	The volume of the upper airway (mm^3^)	2614.81 (1947.46–4752.54)	6108.26 (2968.57–8268.58)	0.012
1- to 3-y age	The narrowest cross-sectional area (mm^2^)	14.96 (13.77–20.69)	21.07 (16.68–29.71)	0.018
	The transverse diameter (mm)	1.95 (1.82–2.91)	2.94 (2.01–3.26)	0.043
	The anteroposterior diameter (mm)	7.36 ± 2.16	8.10 ± 0.82	0.376
	The length of upper airway (mm)	53.05 ± 6.70	58.44 ± 6.40	0.002
	The volume of the upper airway (mm^3^)	5810.81 (3879.34–8173.70)	9071.90 (6696.92–10514.20)	0.018
3- to 5-y age	The narrowest cross-sectional area (mm^2^)	20.16 (19.33–28.17)	38.95 (26.84–43.09)	0.008
	The transverse diameter (mm)	2.74 (2.31–3.09)	3.16 (2.36–4.24)	0.173
	The anteroposterior diameter (mm)	8.21 ± 1.58	10.22 ± 1.58	0.038
	The length of upper airway (mm)	55.57 ± 4.34	62.52 ± 4.80	< 0.001
	The volume of the upper airway (mm^3^)	7441.89 (5525.97–9349.48)	10655.77 (9058.70–11847.39)	0.008

*P* < 0.05 indicates significance

## Discussion

This study explored the impact of lateral positioning on upper airway morphology in sedated children under five. Using 3D reconstruction and Python-based analysis of MRI scans, we measured the narrowest cross-sectional area, minimum transverse diameter, anteroposterior diameter, airway length, and airway volume in both the lateral and supine positions. Our results demonstrated that five the lateral position significantly enlarged these upper airway morphological parameters. The narrowest cross-sectional area, airway length, and airway volume were significantly greater in the lateral position than in the supine position across all age subgroups (0 - 1 year, 1- 3 years, and 3 - 5 years). However, minimal anteroposterior diameter in the 1- to 3-year age group and minimal transverse diameter in the 3- to 5-year age group showed an increasing trend in the lateral position. These changes were not statistically significant. These findings provide a clinical basis for improving airway patency in sedated children.

Adverse events associated with procedural sedation/anesthesia outside the operating room have been reported though these findings primarily originate from small, single-center studies with a wide variance in outcomes, ranging from 2.6% to 82.0% [14, 15]. In Biber’s study, the most common adverse events included persistent desaturation (1.5%), airway obstruction (1.0%), cough (0.9%), and laryngospasm (0.6%) with 1.2% of cases requiring unexpected bag-mask ventilation. Additionally, they reported that younger age groups (< 1 year and 1–5 years) had a higher prevalence of airway-related adverse events [16], likely because of their relatively weaker tolerance to oxygen deprivation. Age is an independent predictor of outcomes during procedural sedation/anesthesia [16]. The unique anatomical characteristics of pediatric patients—including a shorter neck, proportionally larger head-to-body ratio, and a prominent occiput—predispose them to excessive neck flexion. This alignment of the oral, laryngeal, and tracheal axes reduces the anteroposterior diameter and significantly increases the risk of upper airway obstruction in the supine position. Previous studies have demonstrated that when patients are in the supine position, both total airway volume and cross-sectional area are reduced [17, 18]. These morphological airway parameters provide a rapid, intuitive, and safe means of detecting airway obstruction. Therefore, clarification of changes in airway morphology during sedation or anesthesia is crucial for preventing hypoxia in children under sedation.

Sedation required for MRI procedures presents challenges in close monitoring and prompt recognition of adverse events such as vomiting and apnea. Furthermore, the use of certain emergency medical equipment is prohibited within the MRI suite which can complicate the management of any unforeseen complications. In one study analyzing adverse events in sedated pediatric MRI, 22 of 191 patients (11.5%) experienced oxygen desaturation [19]. In another study, unexpected intubation occurred in 2% of 1165 children aged 7 days to 18 years who underwent sedation for MRI scannings [20]. Potentially life-threatening adverse events such as oxygen desaturation and airway obstruction are more pronounced and occur more rapidly in children than in adults [21–24]. Interventions such as chin lift, jaw thrust, and neck extension can alleviate airway obstruction [25]. However, these measures may disrupt the MRI examination process. Our study indicates that the lateral position significantly enlarges the morphology of the upper airway in sedated children under five, thereby helping to alleviate airway obstruction. Compared with other methods, initiating the MRI examination with the patient in the lateral position may be more convenient and efficient. Similar to our findings, previous studies have also demonstrated benefits of lateral positioning in sleeping or anesthetized individuals [26, 27], paralyzed adults under general anesthesia [28], and patients with obstructive sleep apnea [29, 30] or morbid obesity [31, 32]. Furthermore, intraprocedural cough (26.7%) was significantly more frequent in the supinethan the lateral position (10.0%). This is  likely because the lateral position facilitates the clearance of secretions from sensitive midline airway structures. For patients with anticipates difficult airways, intubation in the lateral position required less time to successfully secure the airway [33]. Therefore, lateral positioning is a valuable approach for maintaining ventilation, particularly in spontaneously breathing patients who are lightly sedated for minor surgeries or examinations [28].

Pediatric airways are far from being a smaller version of adult airways. They possess unique anatomical and physiological characteristics, including greater resistance of the upper and lower airway due to narrow spaces and pliable structures, which contribute to the rapid onset of respiratory distress in childhood [1]. Despite routine use children under sedation/anesthesia can develop serious adverse events [7]. Difficult airway management in children is also associated with significant morbidity, particularly in neonates and infants, who are at high risk for complications such as hypoxemia and hypoxic cardiac arrest [34]. Consistent with our findings, Litman et al. reported a significant enlargement of upper airway volume in deeply sedated children aged 2 to 12 years in the lateral position [35]. Studies have shown that adenoids rapidly increase in size from birth to 5 years of age and then gradually regress after the age of 6 years. Length of the upper airway and surrounding tissues grows with age, and the size of the upper airway lumen is primarily influenced by development and regression of the adenoids during growth [35–37]. Children under the age of 5 years have distinct physiological and anatomical characteristics. Therefore, we selected children aged 0 to 5 years of age to explore morphological effects of the lateral decubitus position on their airways. Our findings further support the use of positioning strategies to alleviate upper airway obstruction in younger children, expanding potential clinical application.

In the stratified analysis, significant differences were identified in the volume of the upper airway, the narrowest cross-sectional areas, and the length of the airway across different subgroups. Although there was an increasing trend in minimal transverse and anteroposterior diameters of the cross-section, these changes did not reach statistical significance. This discrepancy may be due to the irregular shape of the most constricted cross-section, where transverse and anteroposterior diameters may not fully capture deformation of the airway. By contrast, 2D and 3D morphological parameters demonstrated consistent stability across all subgroups. Therefore, the use of 3D reconstruction is considered crucial for accurate assessment of these morphological changes.

This study has some limitations. Firstly, there is a risk of selection bias because subjects were retrospectively selected by criteria instead of random assignment. We have  included all eligible cases to reduce this bias. Secondly, sample size is small due to infrequent use of dual-position MRI scannings. Despite modest size, case consistency provided some power. Larger prospective studies are needed for a better evaluation of the lateral position in airway management for younger patients. Thirdly, different levels of sedation land sedation drugs might cause differences in muscle relaxation and respiratory depression, potentially underestimating the role of position in airway patency.

In conclusion, using 3D reconstructions of MRI scans, we demonstrated that the lateral position significantly enlarges the morphology of the upper airway in sedated children under five. This finding provides valuable clinical evidence to ensure airway patency in sedated children and expands the potential application of lateral positioning in alleviating upper airway obstruction. This is particularly relevant in younger children under five.

## Data Availability

The data used and/or analyzed during the present study are available from the corresponding author on reasonable request.
